# Construction Notes

**Published:** 1905-01-28

**Authors:** 


### CONSTRUCTION NOTES.

The latest contributions received at the Bank of England for King Edward's Hospital Fund for London include one for £1,000 from the Worshipful Company of Goldsmiths.

The annual ball of the Royal Isle of Wight Infirmary and County Hospital, which was attended by Princess Henry of Battenberg, president of the hospital, realised £157 10s.

The foundation-stone has been laid of a convalescent home for aged Frenchmen at Brighton. The home will be carried on in connection with the French Hospital in London.

Gratitude being proverbially short-lived, it is interesting to record that St. Thomas's Hospital has received a donation from a former patient in recognition of benefits received in the old hospital nearly fifty years ago.

An isolation hospital, erected at a cost of about £15,000, was opened at Acton last week on the site of a famous private residence. It provides accommodation for 33 beds, five in the observation ward and 14 in each of the other two buildings.

Canon Fleming's sermon "Recognition in Eternity" still continues to earn a substantial sum for the British Home for Incurables. Since 1892, the date of its delivery at Sandringham, £1,600 has been derived from its sale. The Queen allocates the proceeds annually to the Home for Incurables and the Gordon Boys' Home.

Five Liverpool charities—the Royal Northern Hospital, the Royal Southern Hospital, the Royal Infirmary, the Bluecoat Hospital, and the School for the Blind—benefit largely under the will of Mr.

John Lightfoot Newall, of Forest Hall, Ongar, Essex, who died November 29th last year, his estate being of the gross value of upwards of £58,000, and net personality sworn at over £19,000.

THE trustees of the estate of the late Mr. Siegfried Rudolf Zunz have offered to make a grant of £3,000 to the East London Hospital for Children at Shadwell, towards the cost of the whooping-cough wards, etc., now in course of erection, on condition that a ward be named the "Annie Zunz" ward, and that the balance of the money required to complete the building be obtained by June 30th next; £10,000, in all, is still required.

